# Assessment of deep learning algorithms to predict histopathological diagnosis of breast cancer: first Moroccan prospective study on a private dataset

**DOI:** 10.1186/s13104-022-05936-1

**Published:** 2022-02-19

**Authors:** H. El Agouri, M. Azizi, H. El Attar, M. El Khannoussi, A. Ibrahimi, R. Kabbaj, H. Kadiri, S. BekarSabein, S. EchCharif, C. Mounjid, B. El Khannoussi

**Affiliations:** 1 Pathology Department, Oncology National Institute, Faculty of Medicine and Pharmacy, Mohammed V University, 10100 Rabat, Morocco; 2 Datapathology, 20000 Casablanca, Morocco; 3 Anatomic Pathology Laboratory Ennassr, 24000 El Jadida, Morocco; 4 Medical Biotechnology Laboratory (MedBiotech), Bioinova Research Center, Rabat Medical & Pharmacy School, Mohammed Vth University in Rabat, 10100 Rabat, Morocco; 5 Pathology Department, Oncology National Institute, Faculty of Sciences, Mohammed V University, 10100 Rabat, Morocco

**Keywords:** Breast cancer, Digital pathology, Artificial intelligence, Deep learning, Machine learning, Convolutional Neural Networks

## Abstract

**Objective:**

Breast cancer is a critical public health issue and a leading cause of cancer-related deaths among women worldwide. Its early diagnosis and detection can effectively help in increasing the chances of survival rate. For this reason, the diagnosis and classification of breast cancer using Deep learning algorithms have attracted a lot of attention. Therefore, our study aimed to design a computational approach based on deep convolutional neural networks for an efficient classification of breast cancer histopathological images by using our own created dataset. We collected overall 328 digital slides, from 116 of surgical breast specimens diagnosed with invasive breast carcinoma of non-specific type, and referred to the histopathology department of the National Institute of Oncology in Rabat, Morocco. We used two models of deep neural network architectures in order to accurately classify the images into one of three categories: normal tissue-benign lesions, in situ carcinoma or invasive carcinoma.

**Results:**

Both Resnet50 and Xception models achieved comparable results, with a small advantage to Xception extracted features. We reported high degrees of overall correct classification accuracy (88%), and sensitivity (95%) for detection of carcinoma cases, which is important for diagnostic pathology workflow in order to assist pathologists for diagnosing breast cancer with precision. The results of the present study showed that the designed classification model has a good generalization performance in predicting diagnosis of breast cancer, in spite of the limited size of the data. To our knowledge, this approach can be highly compared with other common methods in the automated analysis of breast cancer images reported in literature.

## Introduction

According to the World Health Organization, Breast cancer (BC) constitutes the first major cause of women’s death [1]. In Morocco, 11,747 of women’s new cases with BC were diagnosed during the last year. It represented about 19.8% of all new cancer cases and 38.9% of all cancers in women [2].

Around the world, we are faced with an exponential increase in cancer cases, growing numbers of patients from an aging population, and a shortage of trained pathologists [3]. Moreover, there is a need for accuracy in histopathologic diagnosis of BC as patient demand for accurate diagnostics and personalized therapy is increasing [4, 5]. Therefore, the trend towards digitization of pathology data has opened the door to faster, more precise and more reproducible diagnosis through computerized image analysis [6].

In addition, this will revolutionize the laborious work of the pathologist, which is often repetitive and time consuming, causing significant intra and inter-observer variability [7, 8]. Facing these issues, it is urgent to develop an automatic and an accurate histopathological image analysis methods, especially classification tasks.

Recently, we have witnessed groundbreaking improvements in digital pathology (DP) and artificial intelligence (AI), promising to change the way we detect and treat BC in the near future [9]. The most promising advance in AI is Machine learning (ML), and particularly Deep learning (DL) [10]. In breast pathology, convolutional Neural Networks (CNNs) are favoring deep learning approaches for BC classification and detection [11, 12].

In this paper, we present a classification approach for predicting diagnosis of breast cancer on slide digitized pathology images, using jointly deep CNNs for feature extraction and gradient boosted trees for classification. Then, we discuss the results and compare our framework to several state-of-the-art approaches using similar methods.

## Main text

### Methods

#### Study description

The study was prospectively performed in the histopathology department of the National Institute of Oncology in Rabat, over a period of 6 months from January 2020 to June 2020, involving 116 breast surgical specimens. Only diagnosis of invasive breast carcinoma (IBC) of non specific type was included on all breast surgical specimens. All diagnoses of IBC of a specific type, as well as tumors lysed after neoadjuvant chemotherapy were excluded.

In this study, the tumor tissue samples were stained with hematoxylin–eosin (HE), photographed at 200× equivalent magnification, and exported to jpeg format using *olympus cellsens entry* software. This process was performed by one pathologist, at light microscopy, using *Olympus BX43*, coupled with camera *DX73*. Furthermore, two qualified consultant breast pathologists; completed a brief training in use of the digital microscopy system, were recruited to participate in the validation study.

#### Dataset collection

**Fig. 1:**
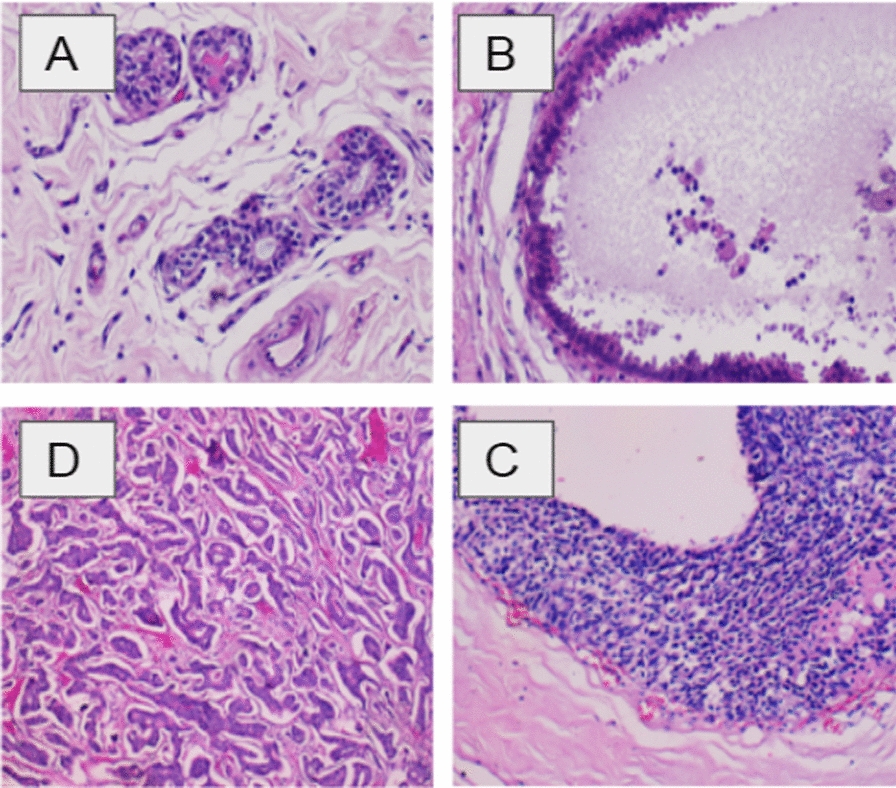
Examples of breast histopathological images in our dataset: **A** normal; **B** benign; **C** in situ carcinoma; and **D** invasive carcinoma (hematoxylin–eosin stain, original magnification ×200)

We collected overall 328 HE stained images. Each image is labeled with one of three classes (Fig. 1): invasive carcinoma (IC) (group 2); in-situ carcinoma (IS-C) (group 1) and benign: benign lesions and or normal tissue (group 0). The labeling was performed by two pathologists, who only provided diagnostic information from the image contents, without specifying the area of interest for the classification.

#### Proposed methodology

When a pathological image with high resolution (2048 × 1536 pixels) is input, our goal is to accurately classify the image into one of three categories: normal or benign, IS-C and IC. To achieve this, we have proposed and tested a method for BC classification inspired from the experimental protocol proposed by Alexander Rakhlin et al. [13]. In our work, each phase is described in the following subsection:

#### Data pre-processing and augmentation

**Fig. 2:**
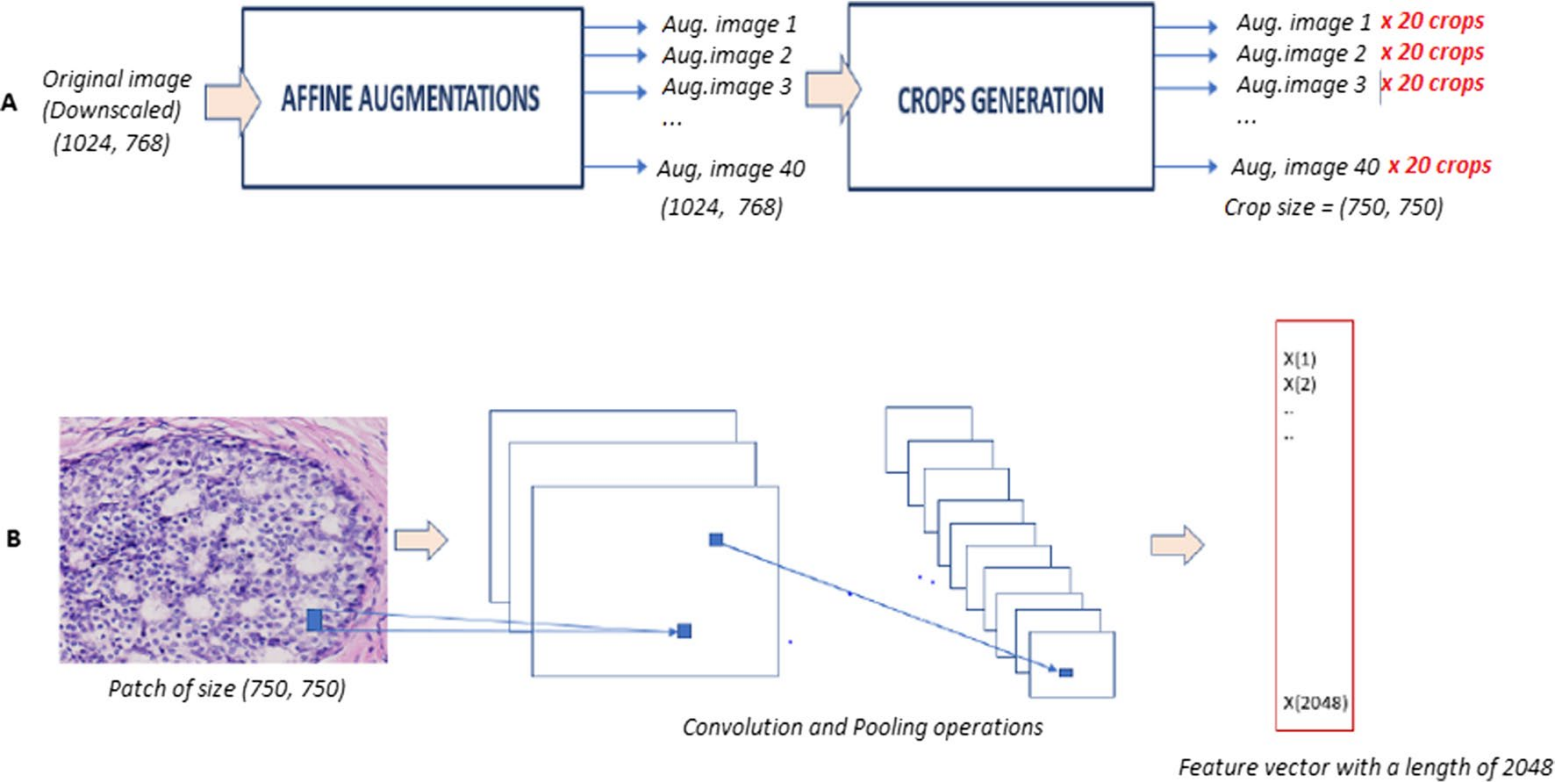
An overview of the proposed methodology. **A** Illustration of data-augmentation: from original image to augmented crops. **B** Illustration of convolutional neural network: from input to output image

Input Dataset is composed of 328 original images, which sized 1024 × 768 at 200× equivalent magnification. Before preforming images augmentation, original images are resized by dividing the initial size in two in order to accelerate the later operations. After a color normalization step, we performed 40 random color augmentations for each image. The augmentation consists of an affine transformation of the input images pixel intensities that allowed us to multiply the size of the dataset by 40. Consequently, each image was used to generate 20 randomly extracted patches of a fixed size (750 × 750), lately processed by the CNNs (Fig. 2A).

#### Features extraction

For our use case, we opted for two Deep CNNs architectures: ResNet50 and Xception models. These two models are pre-trained on the ‘ImageNet’ Dataset, available for public usage, and which contains more than 1 million images or about 150 GB of annotated images of several categories. Both models will be used to compute a descriptor vector for each crop. The feature vectors of the 20 crops of a single image will be combined through a pooling operation to generate one feature vector per image (Fig. 2B).

#### Machine learning classification

We performed a supervised classification using XGBoost model. It is an optimized distributed gradient boosting library, which can be efficiently executed on a GPU station (Graphics Processing Unit), to allow a quick training and evaluation of the model. In fact, gradient boosting models are being extensively used in machine learning due to their speed, accuracy, and robustness against overfitting.

#### Evaluation metrics

To validate our approach, we used a cross-validation method. The augmented images that were extracted from the same original image were placed in the same fold to prevent information leakage. We used a sixfold cross validation strategy, leading to accuracy metric for each fold and then an average global accuracy. Due to a very limited number of images we had for this study, we did not manage to have an additional separate test set. In our work, we computed a prediction for each augmented image then combined the decision made for the 40 augmentations through a voting strategy, in order to compute a unique prediction for each image. In addition, we evaluated the performances for two scenarios, each one corresponding to a CNN architecture of features extraction. We also compared actual class and predicted results obtained using a confusion matrix.

### Results

We have extracted 328 images from HE stained digital slides, among which 152 were non-carcinoma and 176 were carcinoma images. The carcinoma class included images of IC and IS-C while the non-carcinoma class contained images of normal tissues as well as benign lesions. In our study, we performed multi-class classification into three classes: groupe 0 (benign): 152 images, groupe 1 (IS-C): 70 images, and groupe 2 (IC): 106 images. Given the results of this classification system, we computed the corresponding metrics for a binary classification case and a multi-class classification case.

We obtained the following results by performing a sixfolds cross validation approach, training on 273 images and testing on 55 images, during 6 rounds. (Table 1).

**Table 1:** Performance metrics of the Resnet50 and Xception architecture on our dataset

Average accuracy (%)		Confusion matrices					Performance evaluation (%)				
		Actual	Predicted				Metric	Class			
			Group 0	Group 1	Group 2	All		Carcinoma vs. non-carcinoma	Group 0 vs. Group 1	Group 0 vs. Group 2	Group 1 vs. Group 2
Resnet 50 model											
F1	91										
F2	84	Group 0	**142**	4	6	152	Sensitivity	93	92	93	88
F3	85	Group 1	9	**50**	11	70	Specificity	87	94	92	84
F4	80	Group 2	12	9	**85**	106	Precision	88	84	87	90
F5	91	All	163	63	102	328	Accuracy	90	93	92	87
F6	76										
Xception model											
F1	90										
F2	85	Group 0	**144**	4	4	152	Sensitivity	95	93	95	93
F3	81	Group 1	10	**54**	6	70	Specificity	88	93	94	90
F4	87	Group 2	9	6	**91**	106	Precision	89	84	91	93
F5	95	All	163	64	101	328	Accuracy	91	93	94	92
F6	82										

The bold data in the confusions matrices have a significance, It means the number of cases that were correctly predicted in each group

Accuracy: average accuracy for three-classification task, using Resnet50 and Xception models, evaluated over sixfolds via cross-validation

Confusion matrices without normalization using Resnet50 and Xception models: vertical axis—ground truth, horizontal—predictions

Performance evaluation: performance metrics of ResNet50 and Xception models for the binary and 3-class classification

#### ResNet 50 model

The Resnet50 model had correctly predicted 277 out of 328 instances; 142 benign instances were effectively benign, 85 IC were actually invasive, and 50 instances were correctly predicted as IS-C), while 51 cases were misclassified. In terms of 3-class classification, majority voting showed good results, achieving an overall accuracy of 84.5% for three classes.

We also reported that overall accuracy increases when only two classes (non-carcinoma and carcinoma) are considered (84.5 vs. 90%). This indicates that the normal/benign and in situ/invasive classes share similar features. In addition, this proposed model achieved an overall sensitivity of 93% for carcinoma classification, which means that our classifier was very good at detecting cancer.

#### Xception model

For instance, among 152 normal cases, 144 were correctly classified as normal, only 4 were wrongly classified as IC and 4 IS-C were missed. We also noticed that the Xception network achieved a maximum overall accuracy of 88% for three classes, slightly bigger than the Resnet50 model.

In comparison with Resnet50, the Xception model showed high classification results for the binary classifications for all the evaluation metrics, as well as 3-class classifications. Additionally, we reported a high sensitivity (95%) for carcinoma cases, which have a great significance in the diagnostic pathology workflow.

### Discussion

#### DP and AI in breast pathology

The automation of BC diagnosis is essential and requires digitalization of the histological slides using the whole-slide imaging (WSI) system [14], which could assist pathologists to improve the accuracy of diagnostic processes [15].

DP had the potential to transform the way in which pathology services are delivered across the globe. Indeed, it made telepathology consultation between expert pathologists easier [16], provide tools for a more efficient workflow and higher reproducibility [17], especially in challenging situations such as COVID-19 pandemic. The goal of DP is not to take over the pathologist’s work, but to improve accuracy and reduce human error [18].

However, laboratories with integrated DP workflows are still sparse nowadays. In Morocco, as a developing country, we are the first one to introduce AI in routine pathology workflow.

In breast pathology, rapid advances in AI along with the growing DP are a promising approach to meet the urgent need for more accurate detection, classification and prediction [19]. Actually, ML and DL algorithms have been widely successful and showed a high performance in terms of BC diagnosis, prognosis, and response to treatment [20–23].

Moreover, several studies highlights the usefulness of AI in the practice of breast pathology [6, 15]. In term of diagnosis, DL approaches had already been applied to detect malignant breast tumors from benign and normal structures, as well as diagnosis of lymph node metastasis [20]. Other algorithms were developed to assess breast cancer grade (tubular formation, nuclear pleomorphism, mitotic figures) and histologic subtypes. They have been also used for automated biomarker scoring [Ki-67, Oestrogen receptor (ER), progesterone receptor (PR), and human epidermal growth factor receptor (HER2)] [23].

In addition to diagnosis setting, DL methods were used to predict patient prognosis [tumour-infiltrating lymphocytes (TILs), risk of disease recurrence (Oncotype DX)] and response to specific therapy based on the morphological features [22].

#### Comparison with the state-of-the-art

First of all, it is worth mentioning that there are few Moroccan studies who have proposed different approaches [24, 25], performed by biomedical engineers and data scientists, for BC diagnosis using ML on public dataset. Yet to know, our experience is the first one, as pathologists, that successfully assesses AI-algorithms for an automated diagnosis of BC using binary and multi-class classifications in one research work, based on our private single dataset.

The effectiveness of our proposed DL approach can be compared with various state-of-the-art studies used for the classification of BC histopathology images. Most of these studies are based on publicly available dataset [26–30]. Meanwhile, most medical image datasets are usually much smaller because of patient privacy issues and the need for expert annotation and labelling [4]. In our study, we used our own created dataset, which has a limited size compared to public image datasets.

The experimental results showed state-of-the-art testing accuracy for BC detection as compared to existing methods. For instance, for Spanhol et al. [31], the achieved accuracy was approximately 84%. In our work, the overall accuracy is 84% when using ResNet50 and 88% with Xception. In comparison with the previous work, our methods present similar performances, even though our training was performed considering 3 classes. Besides, the used dataset contains approximately 2000 images for the referred magnification, which is a significantly larger training set. Moreover, the previous study images were selected in such a way that only relevant regions for diagnosis were present, while in our case some patches in the training and testing sets may not contain the most relevant information to be correctly classified, which can lower the accuracy in term of classification.

In the work of Araujo et al. [32], authors reported a level of accuracy of 77.8% for 4-class and 83.3% for binary-classification. The sensitivity of their method for cancer cases achieved 95.6%. At the same time, our proposed classification allowed us to obtain a high sensitivity for carcinoma cases, which have a great significance in the diagnostic pathology workflow, as the harm resulting from a false negative (patient remains without diagnosis) is much more detrimental than a false positive (patient undergoes additional procedures and treatments such as chemotherapy). In addition, we achieved a high degree of accuracy i.e. 90% (Resnet 50) and 91% (Xception) for binary-classification tasks.

### Conclusions

In this paper, we proposed a simple and effective method for the classification of HE stained histological BC images in case of very small training data (328 samples). To increase the robustness of the classifier we opted for a hybrid pipeline and used strong data augmentation and deep convolutional features extracted with publicly available pre-trained CNNs. In term of classification task, our results revealed a good discriminatory power either for the differentiation between benign and malignant or to classify their three sub-categories.

## Limitations

Although the presented work has clearly demonstrated the powerful classification capacity of AI-algorithms in term of BC histopathology images, we were challenged by the limited size of the dataset which can leads to overfitting. Therefore, to circumvent this issue we opted for a hybrid pipeline and strong data augmentation.

Currently, we are working on the extension of our dataset with other pathology laboratories as well as detection of invasive BC of a specific type in order to improve the accuracy of classification. Moreover, our project for the implementation of the WSI system is boosting the BC diagnostic workflow. In our future work, we intend to use and evaluate other CNNs pretrained models for the features extraction stage, and extend the application usability to other types of cancer, such as colorectal, lung or prostate cancer.

## Data Availability

The datasets used and/or analysed during the current study are available from the corresponding author on reasonable request.

## Abbreviations

BC: Breast cancer
IBC: Invasive breast cancer
IS-C: In-situ carcinoma
IC: Invasive carcinoma
HE: Hematoxylin and eosin
AI: Artificial intelligence
ML: Machine learning
DL: Deep learning
CNN: Convolutional Neural Networks
WSI: Whole-slide imaging
DP: Digital pathology
ER: Oestrogen receptor
PR: Progesterone receptor
HER2: Human epidermal growth factor receptor
TILs: Tumour-infiltrating lymphocytes
